# Use of Wood in Additive Manufacturing: Review and Future Prospects

**DOI:** 10.3390/polym14061174

**Published:** 2022-03-15

**Authors:** Daša Krapež Tomec, Mirko Kariž

**Affiliations:** Biotechnical Faculty, University of Ljubljana, Jamnikarjeva 101, 1000 Ljubljana, Slovenia; dasa.krapez.tomec@bf.uni-lj.si

**Keywords:** wood, 3D printing, additive manufacturing, opportunities

## Abstract

Polymers filled with natural-based fillers have shown growing demand/interest in recent years, including in additive manufacturing. Like most natural fillers in 3D printing, wood particles serve mainly as a filler that lowers the cost of the printing material due to their low price. However, could wood be used as a main ingredient to affect/improve the properties of 3D-printed parts? Several advantages, such as its reinforcing ability, biodegradability, availability as waste material from other industries, ability to be used in different forms or only in partial components, recycling options or even the use of its undesirable hydromorph-induced dimensional instability for 4D printing, indicate the importance of exploring its use in 3D printing. A review of publications on 3D printing with wood biomass and technologies involving the use of wood particles and components was conducted to identify the possibilities of using wood in additive technologies and their potential.

## 1. Introduction

Consumers, industry and governments are increasingly demanding products made from renewable and sustainable resources that are biodegradable, non-petroleum based, carbon-neutral and pose low risks to the environment, human health and safety [1]. Additive manufacturing technologies are rising technologies that can create opportunities for more sustainable production and the development of competitive strategies in their reference market by creating a more sustainable value chain that is shorter (since digital 3D models can be sent over digital connections and produced on site or close by), smaller, more localized and more collaborative and also have the potential to serve unexplored niche markets [2]. Additive manufacturing (AM), unlike traditional subtractive manufacturing, enables more automated fabrication of products or functional components with complex shapes at low manufacturing costs and low residues. However, the materials still need to be improved to widen the available materials and develop more environmentally friendly materials, since many are petroleum based [3,4].

Another advantage of AM is high material yield, since the product is made by applying material layer by layer in well-defined places, while most conventional manufacturing technologies (sawing, milling, drilling, grinding, etc.) are based on cutting material from a blank, thus creating residues/waste materials.

Wood and other lignocellulosic materials or their components (lignin, cellulose, nanocellulose) could be used in additive manufacturing technologies in several ways and could be one of the leading natural-based materials to help decrease the use of petroleum-based resources and decrease its environmental impact [5]. Primary and secondary wood processing generates significant amounts of wood residues, part of which is used for the production of particleboard and fiberboard, while the larger part is used for energy as a low value-added product. The integration of wood materials into additive manufacturing (AM) is of interest due to their positive environmental impact and improved properties [6]. A significant advantage of using wood with biodegradable or recycled polymers is undoubtedly the ability to use waste materials and recycle them after their end-of-life. With the right combination of polymer, wood particles (size, distribution and content), preparation and additives, a wide range of performance levels can be achieved in compounds. For example, sawdust can serve as a reinforcement for bending and tensile strength [7] or just a cheap filler.

Wood could be an inspiration for 3D-printed wood-like products or its structure an inspiration for 3D-printed construction—wood replicating—micro-tomography (copying the shape/size/distributions of wood cells, its structures and functions in trees) [8] to achieve a better weight–strength ratio of structures.

A review of the use of wood in additive manufacturing was conducted to discover the possibilities in this area.

## 2. Methods

The review article has been prepared based on the study of articles on 3D printing and natural materials collected through various search engines over the past few years, focusing on wood, as we are researching in the field of wood science and technology.

**Figure 1:**
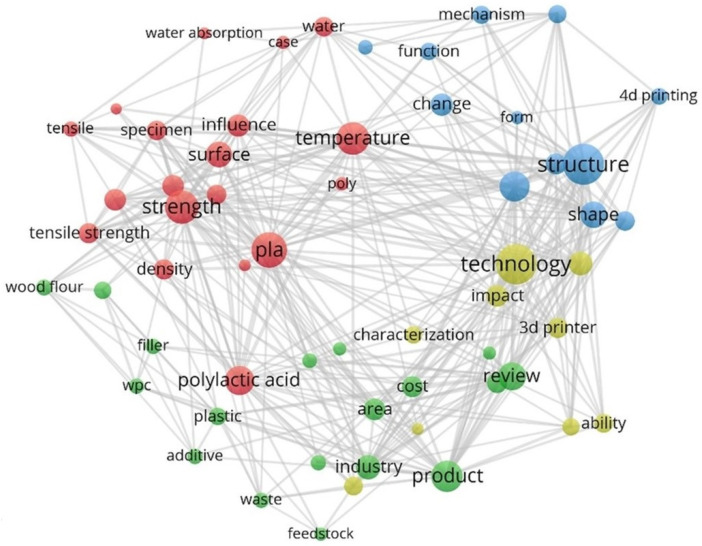
The terms that appeared most in our Mendeley reference database-terms visualization network from VOSviewer v1.6.18 software.

References were collected from our Mendeley database (currently 289 references) and examined. Topics (title, abstract, keywords) were also analysed using the open source software VOSviewer v1.6.18, which helps visualise scientific networks to identify elements that occur in multiple articles and possible connections. Terms with more than 10 occurrences were selected, and the 58 most frequently occurring terms are displayed in four clusters (Figure 1):

- PLA, temperature, strength (red circles)
- Product, review, industry, cost, WPC (green circles)
- Structure, 4D printing, change, reaction (blue circles)
- Technology, layer, impact, 3D printer (yellow circles)

These clusters present the main areas of published articles: The first presents the prominent use of PLA, where temperature and its effect on strength properties are well researched. The second cluster presents topics of WPC, its structure, its use in products and its effect on costs. The third cluster shows the importance of structure, ideas of 4D printing, changing shapes and reactions to exposed conditions. The fourth cluster presents a group of articles dealing with technology, the impact of the used 3D printer and printing parameters.

**Figure 2:**
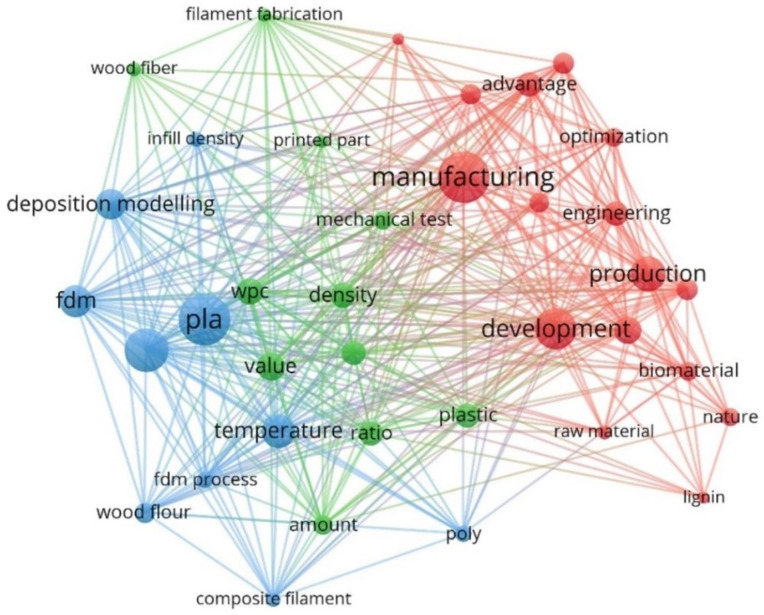
The terms that appeared most from searches concerning our topic in the Scopus database-terms visualization network from VOSviewer v1.6.18 software.

A similar analysis was performed with the search records from Scopus search engine. The search syntax (wood AND (“3d print*” OR “additive manufacturing”) in title, abstract and keywords was used. The subject area was limited to: Engineering, Materials Science, Chemistry, Chemical Engineering and Environmental Science; 333 references were then analysed in VOSviewer v1.6.18 and the following research visualisation map was obtained (Figure 2):

Here, three topic clusters were determined:

- PLA, FDM, deposition modelling (blue circles)
- Density, value, wood fiber, ratio (green circles)
- Manufacturing, development, engineering, optimization (red circles)

Analysis of Scopus search results for this search syntax (wood AND (“3d print*” OR “additive manufacturing”)) shows that the number of publications increased in the last 10 years (from 1 in 2011 to 74 per year in 2021), but this is still small compared to publications on general topics of 3D printing (“3d print*” OR “additive manufacturing”) where the number of documents increased from 346 (in 2011) to 14,206 per year in 2021.

This analysis showed the most researched terms (such as PLA, development, deposition modelling), and helped us determine focus areas, but new and emerging topics/terms are not published enough to be discovered with them. Therefore, the concerns and challenges (Section 5) and future directions (Section 6) are still evaluated from a subjective point of view after studying the literature presented.

## 3. Sources of Wood Material for Use in Additive Manufacturing

Many wood residues come from the wood-processing industry or even biomass from lignocellulosic plants, and wood components (e.g., lignin and tannin as byproducts of the papermaking industry) are often used as an energy source for burning and heating. It has been determined that the use of wood biomass for the production of diverse materials is sensible and increases added value [9]. Moreover, it stores significant amounts of carbon, and using it in products instead of burning it helps to decrease greenhouse emissions [10].

**Figure 3:**
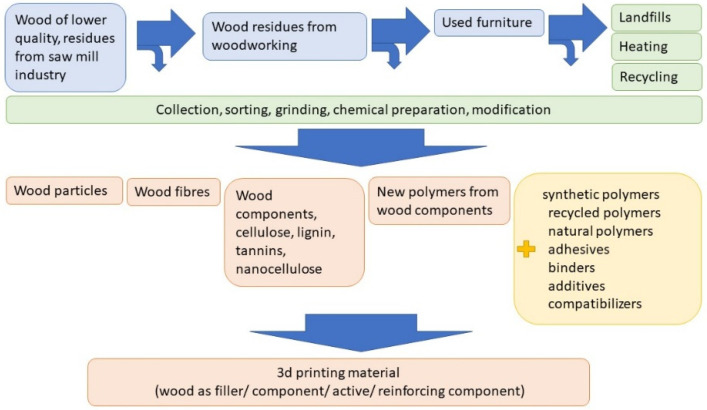
Possible sources for wooden material for use in additive manufacturing.

If processed properly, depending on source origin, some of this biomass could be used as material in various 3D-printing technologies (Figure 3).

Each technology requires different particles and different processing, but the particles are generally ground into particles/fibers of appropriate size and then used as a filler/reinforcing ingredient/component of the printing material. Various fibers/particles or even smaller components of wood such as cellulose, lignin, nanocellulose that need to be chemically prepared are already included in usable printing materials for various techniques. The required degree of mechanical or chemical treatment of the particles depends on the desired properties of the final 3D-printing material. Each treatment requires a large amount of time and energy. Therefore, it would be ideal to use particles that require minimal processing, but this is limited with 3D-printing technologies, for example nozzle diameters. The diameter of the commercial printers nozzle opening is usually 0.2 mm–0.8 mm, thus the particles used need to be smaller than this to prevent clogging. The shape and aspect ratio of particles are also important. Wood particles are randomly shaped and have dimensions that are approximately the same in all directions, while wood fibers have a high aspect ratio (length/width) that gives WPCs good strength. Reinforcement of the polymer matrix with wood fibers significantly improves the mechanical properties of WPCs compared to wood flour at different component ratios [11], but preparation of fibers is more complex.

## 4. Possible Additive Technologies to Be Used with Wood

Several 3D-printing processes differ in the techniques and materials used, but they can be divided into processes that bind the material (binding processes) and processes that deposit material (deposition processes) [12]. The advantage of the bonding process is that, during printing, the entire material layer is applied to the printed surface, which is then bonded (joined) only in the desired areas. The remaining (unbonded) material also serves as a support in the printing phase and can be easily removed after printing (by draining the resin or removing the powder) and used for the next printing. In deposition processes, the material is deposited at precisely defined points in each layer. For more complex products (e.g., overhangs on the product), a support structure must be built up at the same time, which has to be physically removed at the end.

- Great potential for the use of wood in additive technologies has been found in:fused deposition modelling (FDM)/fused filament fabrication (FFF),
- extrusion-based 3D printing,
- inkjet powder printing/droplet application/spraying of binders,
- Laminated Object Manufacturing (LOM),
- Stereolithography (SLA).

Of these methods, FDM (Fused Deposition Modelling) (or FFF (Fused Filament Fabrication)) is the most researched and increasingly appreciated in the last decade and has become the manufacturing method for desktop 3D printers. FDM 3D printers are now affordable and available to DIY enthusiasts. In addition, wood particles are often used in FDM materials, and several wood–polymer filaments are on the market.

The technology for the production of wood–plastic filaments comes from the production of wood–plastic composites (WPC), which have been used for some time, and their production is increasing rapidly. They are mainly known in the form of extruded profiles/sheets used as substitutes for solid wood for floor and wall coverings, fences, door and window profiles, parts of dashboards in cars, packaging, etc. Their production is based on melting thermoplastics mixed with wood particles and extruded through a matrix or in moulds. The polymer matrix serves as a basic structure and transfers external loads to the wood particles/fibers while protecting the particles from external influences [13]; it also acts as a moisture barrier. Therefore, WPCs exhibit advantageous hybrid properties between wood and plastic. Chemically, the plastic matrix binds the WF so that the forces acting on the composites are uniformly transmitted throughout the mass [14].

Wood–polymer filaments are manufactured in various production steps. These include preparing wood particles by grinding them to a specific size, mixing them with polymers and additives, granulating and extruding filaments that can then be used in commercial 3D printers. Because of the pre-processing steps involved in filament production, which include grinding and drying the wood, there is a wide range of wood residues suitable for this application. Sawdust or similar residues from the wood industry can be used as a valuable waste source for the production of high-quality 3D polymer materials. A recent study by Narlıoğlu and co-authors concludes that waste pine sawdust is a suitable reinforcing material for the production of composite filament for 3D-printing applications; it can be extruded with PLA polymer [15] but also other components, wood flour, lignin [16] and cellulose nanofibers, are being explored as functional additives and reinforcements in thermoplastic and thermoset matrices used in additive manufacturing (AM) or 3D printing [1]. The advantages of blending wood into PLA include improved biodegradability, high stiffness and low density compared to pure PLA polymer [17].

**Figure 4:**
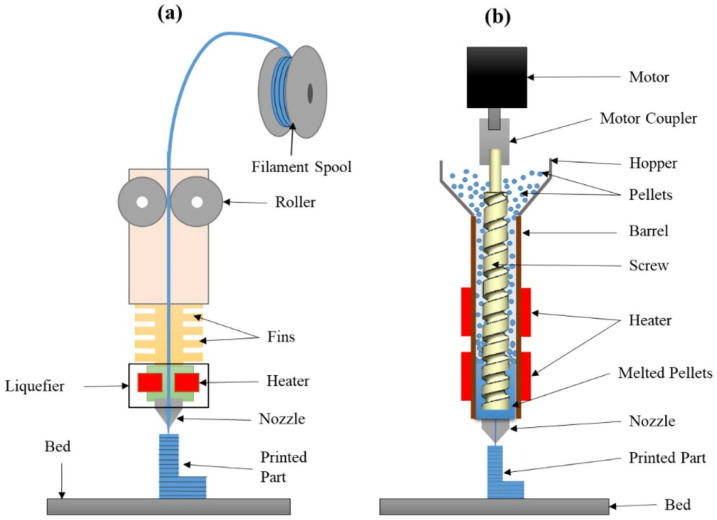
Schematic of (**a**) FFF process and (**b**) pellet extrusion process. Reprinted from [18] with permission from Elsevier.

The filament production step is energy intensive and development toward the use of direct pellet/granules extruders is necessary. Filament prices are quite high compared to pellets, but few pellet extruders are available on the market for smaller 3D printers and have various print quality issues, mainly due to the difficulty of maintaining a stable material flow and removing air bubbles from the molten pellets. The development of pellet extruders (Figure 4) could reduce material costs for 3D printing and open new material sources, such as shredded waste packaging polymers.

Wood can be combined with various polymers, but not all of them are sufficiently compatible with them. One of the main criteria for polymer selection is its melting temperature, as wood should not be exposed to very high temperatures during filament production and printing. Prolonged exposure of wood particles to high temperatures leads to thermal degradation, initially manifested by a change in colour and the appearance of undesirable odours, and the mechanical properties of the particles are reduced [19]. In addition, the formation of gases at higher temperatures leads to a porous structure of the materials, lower density and reduced mechanical properties. Polymers with a softening temperature below 200 °C or below the temperature of thermal degradation of wood particles are suitable. These are mainly polystyrene (PS), polyethylene (PE), polypropylene (PP), polyvinyl chloride (PVC) and other polyolefins [13].

Some of them could be obtained from packaging waste, for which we currently do not have the best reuse strategy, and a large part of which often remains in landfills or even accumulates in nature (oceans). In 2016, 27.1 million tonnes of plastic waste was collected in the EU. Of this, 31.3% was recycled and reused, 41.6% was used for energy, and 27.3% ended up in landfills (Plastics—the Facts 2017). Low-density polyethylene (LDPE) and high-density polyethylene (HDPE) are commonly used for milk bottles, juice bottles, freezer bags, shopping bags, detergent and chemical packaging; polypropylene (PP) is used to make ice cream containers, microwave oven storage containers, squeezable packaging, etc. Most other polymers used for packaging require higher temperatures during processing (220 °C and above), which limits their use with wood particles.

Sometimes, it is also necessary to use higher temperatures for technological reasons; polymers have lower viscosity at higher temperatures, which reduces the energy needed for extrusion (both in filament production and in the printing process). Increasing the proportion of wood particles also increases the viscosity of the composites during production, which implies the use of higher temperatures to reduce the viscosity of the polymers and thus facilitate extrusion [20]. A balance must be found between high temperatures, rheology modifiers and extrusion forces.

For the appropriate properties of wood–plastic composites, it is also necessary to ensure the compatibility of wood particles and polymers or to use additives that allow a suitable bond between the surface of the polymer and the wood. Adhesion between the polymer and the wood particles is an interplay of chemical bonding, diffusion and mechanical anchorage [13] and can be improved with additives or appropriate chemical treatment of the wood particles: Alkaline treatment, acetylation, stearic acid treatment, benzylation, TDI, peroxide treatment, treatment with anhydrides, silanes, isocyanates, plasma [19,21,22,23,24]. Thermal wood modification is one of the possible modifications to reduce the surface polarity of the particles. Reduced surface polarity would allow better compatibility with the normal non-polar surface of the polymers and, despite the lower mechanical properties of the thermally modified particles, the overall properties of the wood–plastic composite could improve [25].

Polylactic acid (PLA) polymer is one of the most commonly used polymers in commercial wood–polymer filaments, with a low glass transition temperature of 60–80 °C, and an endothermic melting peak observed between 140 and 180 °C, which also depends on the additives present and the previous heat treatment of the PLA in the extruder [26], and thermal decomposition in the range of 240–390 °C. It is preferred for 3D-printer applications due to its ease of fabrication, affordable production cost, and biodegradable properties [27]. However, its biodegradable properties only become operational under certain conditions, required for industrial composting in an oxygen-rich environment with high temperatures (58–80 °C), high humidity (>60% moisture) and in the presence of microorganisms (thermophilic bacteria) [28]. Under these conditions, PLA could be degraded by more than 90% in a short time ranging from 30 to 150 days and is converted by microorganisms into CO_2_, H_2_O and compost ingredients [29].

The main disadvantages of PLA are brittleness and low heat resistance, which can be overcome with the use of natural fibers as reinforcement to improve its properties. Natural fibers have many positive effects: Low density, high specific properties, renewability, biodegradability and low cost [30]. The logic for using wood-based components in 3D printing includes improving the mechanical properties, reduced dimensional instability, i.e., part deformation, improved aesthetics, providing an environmentally friendly alternative to carbon or glass-filled polymer matrices and reducing material costs [1]. The content of added wood particles and its properties affect 3D-printed part properties. Kariz and co-authors tested wood-based filaments using PLA with different contents of wood particles from 10% to 50%. Compared to PLA, the filament with 10% wood content exhibited better tensile strength. Furthermore, dynamic mechanical tests of the 3D-printed wood-based material showed a low storage modulus for a high wood content in the filament [31]. The addition of 5% wood flour to PLA changed the microstructure of the material fracture surface, the initial deformation resistance of the composite was enhanced, the starting thermal degradation temperature of the composite was slightly decreased and there were no significant changes in the melting temperature [6]. Higher loading levels of wood-based components can lead to problems with brittleness and breakage of the filament, as well as issues with even printing the wood-filled filament [32]. With higher wood content and larger particles, nozzle blocking occurs, which leads to uneven flow of material and voids in 3D-printed parts. A pellet-fed extruder may be the solution to some of the problems associated with printing a high fiber content thermoplastic composite. Yang showed that the mechanical properties of PLA with a wood fiber filler were negatively affected at printing temperatures above 200 °C, while density and most physical properties of the printed composite, increased with the increase of printing temperature in the range of 200–230 °C [27].

The study by Guessasma and co-authors (2019) concludes that higher printing temperatures above 230 °C are not suitable for wood–PLA, as tensile properties may be affected by thermal degradation of the wood particles, which occurs between 210 °C and 370 °C. The FDM processing of the wood-based filament results in a complex microstructure characterised by the presence of two different types of porosity: One inherent to the filament and one process induced [33], probably due to gases produced at extruding at high temperatures.

Three-dimensional-printed PLA produced by the FDM process has inferior mechanical properties compared to compression-molded PLA. The tensile strength and modulus of 3D-printed PLA can be improved by 84% and 63%, respectively, by adding 1 wt% cellulose nanofibers (CNFs), making it comparable to compression molded samples. The improved mechanical properties of the 3D-printed PLA/CNF composites can be attributed to higher crystallinity and lower porosity [34]. Three-dimensional-printed high-performance cellular composites made from wood fiber-reinforced PLA (WF-PLA) offer a unique strategy for additive manufacturing sustainable advanced materials with improved thermomechanical properties from low-cost waste materials through optimized material composition and rational design [35].

Lignin is an extensive and inexpensive raw material that could replace some synthetic polymers. It is the second most abundantly available natural polymer after cellulose and is the glue that binds cellulose fibers together and provides stiffness in plants. As a byproduct of pulping in the paper industry, it has yet to attract high-value niche applications despite its perceived potential. Depending on biomass source and processing routes, different strains of lignin are produced, such as alkaline lignin, kraft lignin, organosolv lignin and others [16]. The inherent properties of lignin (i.e., antioxidant and antibacterial properties) but also its tendency to revert to humus after biodegradation can be expected to be preserved in lignin-based parts [36].

The research of compatibility of three different types of lignin (i.e., kraft lignin, organosolv lignin and lignosulfonate) with PLA, especially without the addition of other reagents or compatibilizers, showed poor mechanical properties for the kraft lignin–PLA blends but a higher compatibility of organosolv lignin–PLA blends. Furthermore, lignosulfonate–PLA blends showed promising behavior for 3D printing. A specific nucleation effect was observed in this study, which could be useful for foaming applications [37].

Wood particles could also be used in binder jetting [38], where wood powder could be mixed with commercial ZP102 material from the Z Corporation or other raw materials [39], where binder (gypsum, cellulose, sodium silicate and cement) was mixed with wood in a dry state and water was sprayed on as an activator. Since grinding wood into fine particles is energy and time consuming, other sources of wood particles are being researched, e.g., wood particles from house borers and drywood termite frass [40].

Wood-based inks for 3D printing were developed by Markstedt and co-authors with a biomimetic approach that exploited the structural properties of cellulose and the cross-linking function of hemicelluloses found in the plant cell wall. The cellulose nanofibrils were mixed with xylan, a hemicellulose derived from spruce wood, to introduce the crosslinking properties that are critical to the final stability of the printed ink. The wood-based ink not only meets printability and crosslinking requirements but is also a sustainable choice for future materials to be used for the 3D printing of clothing, packaging, healthcare products and furniture [41].

Dry wood powder can be mixed with polypropylene or copolyester hot melt adhesives and used with Selective Laser Sintering (SLS) technology [42]. The mixture was spread in thin layers, 0.1–0.2 mm, and sintered at selected places with CO_2_ laser using a low power, 7–14 W. Sintered WPC parts were porous and needed to be post-treated with wax or epoxy resin to improve their performance. To achieve better adhesion of wood flour and polymers, the wood particles could be modified via alkalinization and adding compatibilizer. The test results show alkaline treatment decreased the hydrophilicity of wood flour, increased the contact area between PES and wood flour and improved the bonding strength. Adding the compatibilizer polypropylene grafting maleic anhydride (PP-g-MAH) can form bonds between the wood flour PES and PP which improves their compatibility and mechanical property more [43].

Modified lignin could be used in photopolymer resins in a commercial stereolithography system. Sutton et al. fabricated resins with up to 15% of lignin, which exhibit excellent printing quality, good layer fusion, high surface definition and visual clarity. Moreover, the ductility of such printed parts increased with higher lignin concentration [44]. The incorporation of softwood kraft lignin in smaller concentrations (0.2 to 1%) in methacrylate resin increased the tensile strength by 46–64% and Young’s modulus by 13–37% for the post-cured printed composites compared with that of the control sample (no lignin added) [45]. These materials can be used to generate new products for additive manufacturing applications and help fill vacant material property spaces, where ductility, sustainability, and application costs are critical.

**Figure 5:**
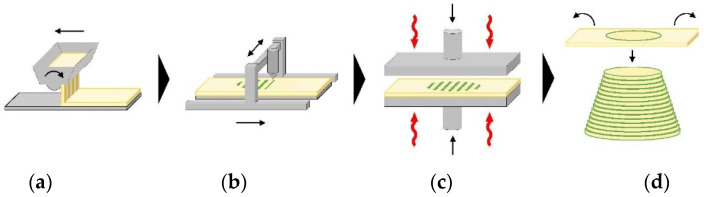
Principle of ILF fabrication [46]. Particles are spread in a thin layer by a scattering device (**a**), a liquid adhesive is applied locally, (**b**). The particle adhesive layer is then pressed and heated (**c**), the unbound bulk is removed, and the completed panel is transferred and laminated onto the stack of the previously fabricated panels (**d**).

Individual layer fabrication (ILF) is a novel AM process designed for structural applications [46]. In ILF, parts are formed by laminating thin, individually contoured panels of wood composites that are fabricated additively by binder jetting (Figure 5). The custom fabrication of individual panels enables the application of mechanical pressure in each layer and the production of these board-like elements, which are then stacked to the final product. The application of pressure increases the density and strength of the individual panel layers and results in a reduction in the amount of binder required. Recent research indicates that individual panels showed good bonding and an acceptable degree of shape accuracy. By laminating a series of panels, a solid object could be created in the desired shape. Challenges were noted with regard to adhesive penetration into the bulk and stacking of the individual panels [47].

Wood could be used with laminating object manufacturing (LOM) technology in various forms as panel plywood or veneers, achieving even higher accuracy [48]. Specially cut and laminated veneers could be used to maintain the strength of wood and also retain the advantageous qualities of AM, specifically, the ability to produce products with complex geometries that would otherwise be impossible with subtractive manufacturing techniques. By adjusting the orientation of the layers, removing wood defects and incorporating other materials, even higher strengths could be obtained. Furthermore, even wooden chopsticks and glue could be used in a kind of human-assisted additive manufacturing [49].

Wood particles or components could also be used in liquid deposition modelling, in which specimens are printed using different paste-like suspensions made from ground beech sawdust and methylcellulose dissolved in water [50]. The wood content could be increased up to 89% in dry mass and affect the physical properties of printed samples. Moreover, paste-like mixtures from wood and commercial wood adhesive could also be used [51] but with only lower percentages of wood (up to 25% of wood in mixture). Higher content of wood often increases the force needed for extrusion through relatively small diameter nozzles, increases nozzle blocking and reduces print quality. Solutions for this could be in rheology modifiers to improve material flow or new technologies developed like vibration-assisted 3D printing, which is used in clay printing [52].

## 5. Applications of Wood Using 3D/4D Printing

### 5.1. Wood in 3D Printing Constructions/Buildings

The use of wood in smaller commercial 3D printers requires significant amount of particle processing to achieve the extrusion of sufficiently small particles through low diameter printing nozzles. If a similar approach could be used in construction, where the required dimensions and accuracy are at a different level, larger nozzles and thus larger particles could be used. This opens new possibilities for the direct use of cheap wood residues from dust extraction systems in the woodworking industry. With a larger building size and larger particles needed (energy and time consuming), milling and grinding particles to smaller sizes is no longer necessary.

Large-scale printers FDM typically have a build volume of 1 cubic metre or more, and these systems use a pellet-fed extruder. The presence of wood- and lignocellulosic-based fillers can lead to poor adhesion because the polymers do not intertwine, problems with crystallisation occur and the dispersion is heterogeneous. To overcome these problems, large-scale printers rely on adjustable screws during extrusion, heated print beds, dual print heads and environmental control, as well as extensive trials with test prints [53]. Recently, AM processes have been explored on a large scale with build volumes of 90 cubic metres and more, covering the product range of automotive manufacturing and construction applications. In addition, the AM of wood and lignocellulosic-based composites has led to improved material properties for a variety of product applications, making them even more attractive [8].

The development of low-cost lignocellulosic-filled thermoplastic composite formulations for use in the large-scale 3D printing of low-cost marine composites tooling was investigated. Wood and lignocellulosic fiber-filled thermoplastics are priced at about 10% of carbon fiber-filled acrylonitrile butadiene styrene (ABS) plastic, which is the most commonly used feedstock for large-scale 3D printing. The use of large scale 3D printing to produce marine tooling provides a more efficient process for manufacturing and final machining and coating, which reduces lead times for tooling production and enables their recyclability [54].

Dawod and co-authors have discovered a novel AM process that uses a continuous solid wood filament to fabricate load-bearing structural elements that provide the micro-scale aesthetics and structural benefits of wood. Split willow withies were used as the raw material for the production of solid wood filaments, as they are highly flexible [55].

Researchers [56] have successfully used poplar to reinforce PLA to obtain bio-based composites suitable for large-scale 3D printing. The tensile strength of the composites increased from 34 to 54 MPa when the poplar fiber size decreased. The decrease in fiber size promoted better dispersion in PLA and allowed better access to surface porosity for PLA penetration [56].

Productivity in the construction industry has stagnated for decades, as manual techniques tend to favour simple component design and thus inefficient use of materials. Moreover, due to the substantial resource requirements in the construction industry, these construction methods contribute significantly to global CO_2_ emissions. While additive manufacturing technology is being used for mass production in other industries, fundamental challenges remain to be solved when transferring it to the construction industry. These include the transfer of AM technologies to the large scale of construction, the necessary diversity of materials and processes determined by the complex functional requirements of a building, meeting the building codes requirements, long term use and the high degree of customisation and flexibility required in construction.

### 5.2. Continuous Fibre Printing

One option to increase the mechanical properties of 3D-printed parts is the use of continuous fibers in printing material. Continuous fiber printing could be achieved with synthetic fibers or natural non-processed materials [57], such as twisted yarns of natural jute fibers. Le Duigou et al. presented a method for preparing continuous flax fiber/PLA composite filament made with a customized co-extrusion process, used for 3D printing with a simple and affordable 3D printer [58].

**Figure 6:**
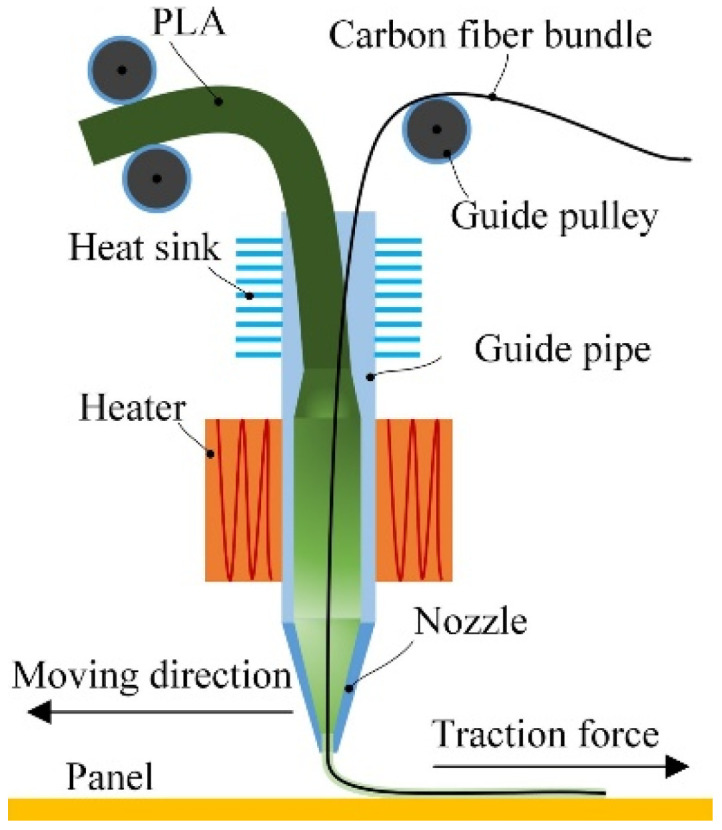
Principle of extrusion device to printing continuous carbon fiber-reinforced PLA. Reprinted from [59] with permission from Elsevier.

Printing with PLA with synthetic continuous carbon fibers is better known and researched Li and co-authors (Figure 6). The nozzle was designed to mix the carbon fibers and PLA resin uniformly. Continuous PLA wire and carbon fiber bundles mix in the guide tube by heating the PLA resin to melting temperature. Considering the weak bond between the carbon fiber and PLA resin, the pre-processing of the carbon fibers was carried out to improve the interfacial strength. The modified carbon fiber printed PLA sample has much higher flexural strength than the original carbon fiber printed sample, and the modified sample has an improvement of about 164%. This rapid prototyping technology for the continuous carbon fiber composite has potential for producing complex and high-performance composite parts, especially for complex aircraft structures [59].

Carbon fiber, a strong and lightweight material, has many potential applications but high production cost, with the raw material and spinning of the fibers accounting for about 50% of the cost. Continuous carbon fibers could also be prepared from wood-based materials. Lignin has a high carbon content (60–65%), indicating a high yield after carbon fiber processing (CF), making it an interesting alternative to petroleum-based polyactrylonitrile (PAN). However, the mechanical properties of the lignin-based CFs produced to date do not yet meet the targets set by the automotive industry [60]. Bengtsson and co-authors claim that the preparation of CFs with the dry jet wet spinning of unfractionated softwood kraft lignin and paper-grade softwood kraft pulp blends is a promising route for the production of cost-effective CFs. Previously, in research by Norberg, carbon fibers were made from kraft lignin derived from both softwood and hardwood. Kraft lignin fibers from softwood can be oxidatively stabilized 26 times faster than hardwood kraft lignin fibers. Moreover, stabilization and carbonization in a nitrogen atmosphere were successfully carried out in a single step. This result opens up the possibility of omitting the stabilization step in the carbon fiber process by using softwood kraft lignin as a precursor [61].

### 5.3. Three-Dimensional Printing and Furniture Design

Small elements used in furniture, such as versatile connector fittings or fasteners for simple connections of shelves and panels, can provide aesthetic dimensions in addition to functional and structural properties. These elements are particularly well suited to manufacturing through 3D printing, which allows for custom shapes and sizes in small batches. The rapid manufacturing and infinite possibilities for quick changes in element design make it very easy to assemble simple wooden boards into functional shelves in a short time, which is particularly important for various mobile furniture solutions. The flexibility and cost efficiency of such small series offer small companies the opportunity to enter new production without significant investment [62].

Three-dimensional-printed connections are convenient when quick disassembly is required, to reduce the product’s weight, or for greater variability of objects or colours. The products then function like a construction kit that can be easily and quickly assembled and disassembled or transformed into another variant. Verifying the mechanical properties of products made with 3D-printing technology is very important to ensure that functionality can be fulfilled [63].

Nevertheless, 3D printing of biomass-filled PLA gives furniture and architectural models a wood-like appearance. Furthermore, it was noted that wood–PLA furniture has excellent scratch and wear resistance; thus, useful household items can be printed with this material [64].

**Figure 7:**
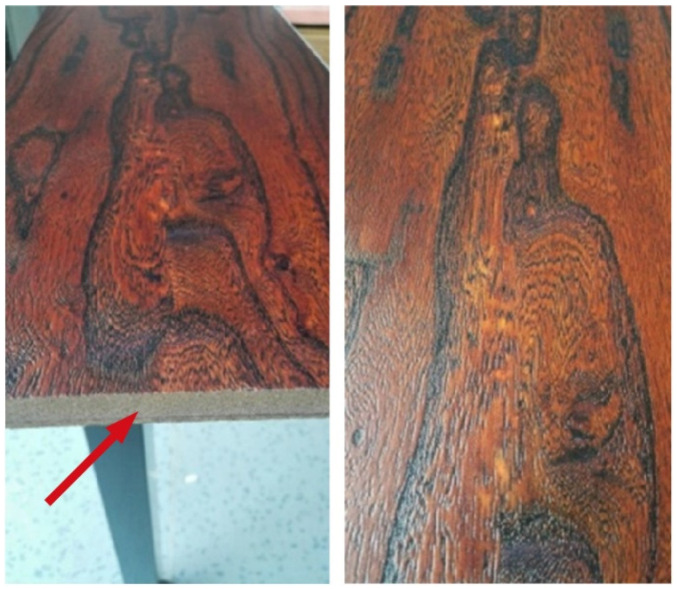
Elm texture 3D printed on medium-density fiberboard (MDF) substrate [66].

There is yet another use of wood in connection with additive manufacturing: The imitation of its texture for decorative purposes (Figure 7). Wood textures of various natural woods are scanned, converted to a UV inkjet print file, and then coatings composed primarily of acrylated oligomers mimicking wood texture are successfully 3D printed on medium density fiberboard using UV inkjet printing [65,66].

A study by Huang and co-authors describes how sawdust can be upgraded as a component of wood–plastic composites (WPC) to produce 3D-printed elements suitable for architectural applications. Wood particles with rounder shapes and smoother surfaces increase the strength, stiffness and density of 3D-printed parts due to the better adhesion of these shaped wood particles with the polymer. A higher ratio of wood to plastic results in a ‘greener’ product with improved stiffness. The ‘double extrusion process’ has been identified as an effective filament processing technique to improve the quality of WPC filaments, leading to a substantial reduction in porosity and a much-improved homogeneity [67].

Another option is mycelium-based composites, which could provide a sustainable alternative to current composites used in architecture. Soh et al. [68] developed a mycelium-based extrudable paste, based on agricultural waste materials, bamboo microfibers, chitosan, and the mycelium of *Ganoderma lucidum*, to be used as a material for 3D printing. A similar principle could be used for the production of furniture [69], sound insulation panels [70] or even bigger structures [71].

### 5.4. Four-Dimensional Printing

**Figure 8:**
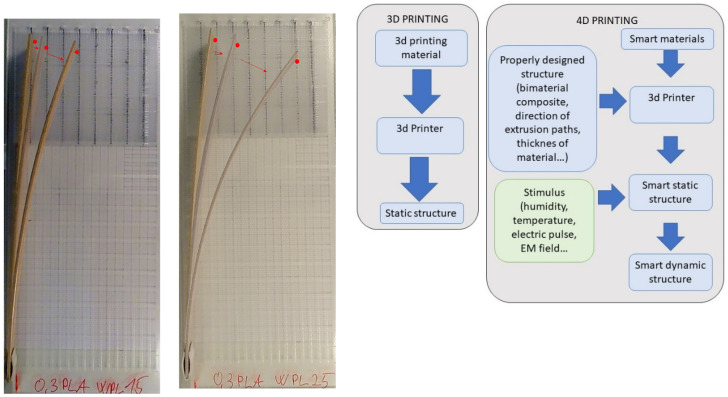
Use of wood–PLA filament in 4D printing—bimaterial actuator principle—elements with layers of PLA and layers of wood–PLA changing shape when exposed to changing climate conditions (**left**) [76]; principle of 4D printing (**right**).

Wood is a natural hygroscopic material that absorbs or desorbs water from its environment under changing climatic conditions, thus changing its dimensions. Since wood retains its hygroscopic character even after incorporation into wood–plastic composites (to a lesser extent, depending on the ratio in the composite and the additives), it could be used as an active shape-changing material in 4D printing. Four-dimensional printing has evolved from 3D printing and aims to achieve a predictable and predefined time-dependent change in functionality (shape, property, self-assembly, or self-repair) that the 3D-printed structure undergoes when it encounters an external stimulus (e.g., temperature, ultraviolet light, humidity, electric and magnetic fields) [72] (Figure 8, right).

In contrast to manipulating natural wood grain, 3D-printing technologies enable the design of specific wood grain patterns to precisely control the direction of warping. In addition, the use of multi-material printing, which combines different materials with wood, can create customised wood composites that produce new macro-scale behaviour. These composites take advantage of the natural expansion and contraction properties of wood, enhancing its transformation properties, and providing greater control over the desired curvature [73].

Actuation in response to a stimulus through pre-programmed hierarchical structures may provide a biologically inspired model useful for functional gradation of natural fibers to evolve hygroscopically induced shape change (i.e., hygromorphing) [74]. Moisture-induced shape-changing bilayer actuators could serve as a principle for active façade or ventilation valves. Bilayer systems with wood particles are particularly well suited for driving external convertible elements because the daily change in relative humidity due to solar energy remains a source of energy, and activation continues despite weathering (or natural ageing of the material) [75].

The disadvantages of wood (dimensional changes due to water adsorption and desorption) could be used as features when dimensional change is desired, such as in shape-changing 4D printed materials. A recent study showed that the shape change depends on the ratio of the materials in the two-layer actuator and the amount of wood in the wood–PLA composite used, and thus on the sorption. The rate of shape change is claimed to react in the same way: the higher the wood content, the greater the observed change. The dynamics of the hygromorphism of bimaterial composites is greater when a small amount of hygromechanically active material is added [76] (Figure 8, left).

The advantage of 4D printing is the ability to encode the embedded parameters through the printing process of wooden responsive actuators as accurate, programmed motion mechanisms. When focusing specifically on wood grain orientation, the printing process controls grain orientation and enable the production of a variety of motion types with single- and double-curved surfaces when exposed to differences in humidity levels [77].

Most research in this area has focused on exploiting hygroscopically induced shape change in wood–polymer materials. There is still much to be explored about proper design, fiber orientation in the extruded material and model and ways to incorporate artificial stimuli into the design, such as heating, to increase the speed of response to stimuli.

### 5.5. Three-Dimensional-Printed Wood-like Products/Biomimicry/Replicating Wood Structure

**Figure 9:**
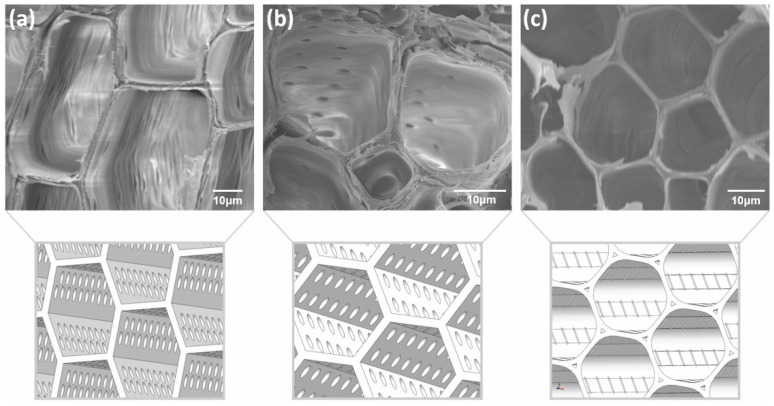
SEM images of wood structure of different wood species ((**a**) cedar, (**b**) oak, and (**c**) palm) and 3D models constructed based on these structures. Reprinted from [80] with permission from Elsevier.

As a result of the rapid development of 3D-printing technologies, more bioinspired structures can now be designed and manufactured. As tomographic methods improve in resolution and computation capacity, it became easier to scan and then 3D print with these systems. Three-dimensional printing could thus be used to facilitate the self-replication of wooden cell structures at various scales of magnification [8]. Biomimicry from macroscopic to microscopic scale is leading the way in the development of high-performance materials and structures. In general, bioinspired structures are always complex, but thanks to the flexibility of 3D-printing technologies, structures with any geometry can be fabricated [78,79]. The main utility of cellular structures lies in their ability to meet performance targets while enabling significant mass reduction, a principle commonly embodied in nature. Ufodike and co-authors designed and FFF 3D-printed novel biomorphic cellular structures inspired by the microstructures of cedar, oak and palm wood. The mechanical behavior and energy absorption capabilities of designed structures (Figure 9) have been studied, and the results suggest that wood microstructures could serve as good model for rational design of additively manufactured porous biomorphic materials and help to decrease material consumption at adequate mechanical properties [80].

Different wood tissues have different structures, adapted to tasks; thus, it makes sense to mimic the most significant wood cell structures. Balsa wood (*Ochroma pyramidale*) has remarkable mechanical properties for its weight; its specific Young’s modulus is even comparable to some engineering fiber composites, as well as its superior energy absorption [79]. Additive manufactured cellular structures, based on mimicking balsa structure, exhibit good mechanical properties, energy absorption, vibration damping and insulation applications [79], but the additive manufacturing of such structures remains limited by poor commercial materials and the lack of effective design tools [81].

A study by Zorzetto and Ruffoni combined 3D polyjet printing and computer simulations to investigate wood-inspired helix-reinforced cylinders. They mimic the multilayered, tubular structure of wood’s cell walls, where each layer has a compliant matrix reinforced by stiff helicoidal microfibrils. The helicoidal biocomposites were arranged in multiple layers with different fiber orientations that improve the mechanical performance and tune the local load bearing behaviour. The study showed how the mechanical functions of synthetic structures can be programmed by varying the fiber/fibril orientation and that fracture strength can be increased by surrounding the main helicoidal layer with a minimum of thin fibrils oriented perpendicularly to the applied load, as observed in wood. These structures have the potential to be used in larger systems, resulting in graded composites with region-specific properties, optimised for multiple applications [82].

In recent years, startups have emerged that use byproducts from the wood industry for the process that can print material with a grain pattern and mimic different wood species, from ash to mahogany. The process involves applying thin layers of sawdust and uses inkjets to print a non-toxic binder (including lignin) to replicate the wood’s grain layer by layer. Unlike particle board or laminate, the grain goes completely through the material; therefore, it can be sanded and post-processed like solid wood. A product such as a chair or a bowl can be printed in its finished form, with no waste and with detailed shapes.

## 6. Concerns and Challenges

As the popularity of 3D printing increases, so does the total number of prints and associated waste material, mainly from support structures. Despite the ease of using PLA in the 3D-printing process, it creates waste that must be disposed of or recycled. Keeping 3D-printing waste out of landfills and reusing it are high priorities. A study by Anderson [83] evaluated the physical properties of objects printed with virgin PLA versus recycled PLA and concluded that using recycled PLA for 3D printing is a viable option.

The environmental impact of 3D printing from energy consumption view could be significantly improved in the design/printing phase by selecting appropriate materials, lowering the printing times and thus lowering energy consumption and proper product design [84].

Since PLA is a highly hygroscopic polymer that is very sensitive to high temperatures (around 200 °C), its outdoor durability is low due to the synergistic effects of air humidity, temperature and UV irradiation. The latter makes PLA not suitable for all applications [85].

Typically, in WPCs, water molecules interact with the cellulose component of the wood fiber and accumulate in the spaces between the wood microfibrils, causing the fibers to swell and the polymer matrix to crack, which results in poor stress transfer efficiency. Moisture absorption of natural fibers affects their physical, mechanical and thermal properties [86]. In most cases, the effects of moisture-induced damage are irreversible, and mechanical performance is not restored after drying, resulting in loss of mechanical properties and dimensional changes due to moisture-induced damage.

Nevertheless, using filaments that are biodegradable is one way to make 3D printing more sustainable and reducing its environmental impact. Biodegradable plastics have often lower mechanical properties and durability, so these materials are best suited for applications with a short life cycle [87]. For example, the research by Gardan and co-authors [88] aimed to produce a reconstituted wood product with low environmental impact by mixing beech flour with chemically modified starch. Composite, produced by cold extrusion—Dough Deposition Modeling (DDM)—shows weak tensile strength and fragile behavior compared to the tensile strength of a polypropylene composite filled with 40% wood flour. The disadvantages are also the sensitivity of the material to environmental conditions with its moisture absorption due to the hydrophilic properties of hemicellulose and starch [88].

However, wood–polymer filaments made from used wood have some concerns regarding their biodegradability. Recycling fiber mixed composites requires more effort than recycling pure polymers. One recent study even advises caution about biodegradable polymers since large amounts of microplastics from biodegradable polyester were formed during enzymatic hydrolysis in aqueous environment [89]. However, a study by Fortunati and co-authors showed that the addition of 10–30% biomass fibers to PLA notably reduced the biodegradation time of the composite [90], which is promising information for wood–plastic filaments.

Regarding the use of waste/used wood, it is also often difficult to distinguish between pure wood and wood residues treated with chemical preservatives (e.g., waste wood treated with older versions of preservatives, e.g., chromated copper arsenate, which is now considered dangerous waste) or panels with formaldehyde adhesives, which could cause serious health concerns due to their adverse effect on the environment. Furthermore, engineered wood products made by using wood fiber and a binder are not completely biodegradable due to the use of nonbiodegradable binders such as urea-formaldehyde (UF), phenol-formaldehyde (PF), melamine-formaldehyde (MF) and methylene diphenyl diisocyanate (MDI) or ethyl carbonate (urethane) resins [91].

A study by Pringle and co-authors examined the possibility of upcycling furniture waste as a feedstock for filaments. According to the authors, the furniture industry in Michigan alone produces more than 150 tons of wood waste per day. The wood waste (solid slabs and sawdust from MDF, LDF and melamine) was hammer milled and ground into a powder to make the wood mixable with PLA. Batches containing 30 wt% wood proved to be the most promising in terms of usability [92], but no environment effects were considered.

Industrial processing of thermoplastics at temperatures of 180–280 °C, where printing is mainly done, leads to undesirable emissions of volatile and semi-volatile organic compounds and particles. The most critical period seems to be the beginning of the printing process, when the particles emitted are usually at their highest concentration and smallest size [93]. Reducing the nozzle temperature, selecting a material that operates at a lower nozzle temperature and using lower-emission filaments (e.g., PLA filaments, which have low ultrafine particles (UFP) [94]) can reduce emissions of volatile organic compounds and ultrafine particles [95].

Analysis of the formation of volatile organic compounds (VOCs) from lignocellulose/PLA filaments showed that particle formation dominated during the heating process, while VOCs were mainly released during the printing process. Printing at higher relative humidity and high filament feeding temperatures resulted in higher VOC emissions. In addition, high humidity facilitated particle agglomeration and reduced particulate matter (PM) concentration. When the nozzle of the FFF 3D printer was clogging, the particle matter concentration increased (actually doubled) [96].

The major VOCs compounds emitted from lignocellulose and PLA filaments are acetyl tributyl citrate, tributylprop-1-ene-1,2,3-tricarboxylate and L-lactide. Therefore, indoor air quality must be closely monitored, and it is strongly recommended that the 3D printer be used only in a well-ventilated environment [96]. Nevertheless, the development of ventilation options and guidance for the safe use of 3D printers in various environments, including a low-cost exhaust hood (equipped with a high-efficiency particulate air (HEPA) filter and an activated carbon filter), should be a priority [97].

## 7. Discussion

Based on the literature review, we summarise that wood could be used in 3D printing in several ways. The most promising 3D-printing technologies and applications associated with the use of wood are summarised in Table 1.

As shown in Table 1, the most researched AM processes are extrusion, granular material bonding and liquid deposition modelling. The percentage of wood or wood components is typically 10–40%, but some technologies use an even higher percentage of wood. The highest wood content, up to 89% in dry mass, was used in samples printed by liquid deposition modelling technology. Therefore, increasing the wood content in printed materials is the next goal of research in this area. When using higher wood contents, issues related to the use of compatibilizers, or different modification techniques need to be addressed to ensure better blending of wood with polymer and smoothing of the surface in post-processing.

In extrusion processes for thermoplastics and composites, the feedstock is usually in the form of filaments (e.g., FDM process) and less frequently in the form of pellets/granules (e.g., large-scale platforms). The development of pellet extruders (also for smaller printers) would lower the price of printed products, as polymer pellets are cheaper than filaments. Optimized pellet extruders with higher material flow and the possible use of printing nozzles with larger diameters would open the possibility of using materials with larger wood particles/fibers and printing larger objects.

Consumers, industry and governments are increasingly demanding products made from renewable and sustainable resources that are biodegradable, non-petroleum based, carbon neutral and pose low risks to the environment, human health and safety [1]. This could be the future driving force behind the increased use of wood and its components in 3D printing. In this regard, it is worth encouraging life cycle assessment (LCA) analysis, to address environmental impact of composite materials and products. In order to improve disposal alternatives and minimize environmental impacts, it is important to study impacts related to emissions and energy consumption, land use, and genotoxicity of residues [103]. Huge impact can be made on environmental impact of product in the design phase by selecting proper design (less material used, lower mass for transportation, less waste, better efficiency), proper materials (suitable mechanical properties, material’s origin, etc.) and production technology (time, energy consumption).

Challenges in additive manufacturing with wood composites include processing issues during extrusion and part fabrication, particularly with regard to the dimensional stability of parts and brittleness of the material depending on the loading level of the wood components, as well as effects on the crystallisation behaviour of the polymer during processing [1]. In certain cases, printed parts’ mechanical and physical properties can approach the property range of conventional wood composites such as particleboard, fiberboard and wood thermoplastic composites. With proper incorporation of wood fibers, nanocellulose and continuous fiber printing, this could be even improved.

Lower interlayer (tensile) strength is still a problem in 3D printing, even when using wood–polymer based materials, especially when printing with higher layer thicknesses. With the goal of using wood–plastic composites for larger objects or even structures, further research is needed in this direction by exploring options for optimizing build orientation, post-processing, use of multi-axis 3D printing, heating methods prior to deposition of each layer, application of pressure, etc., [104].

Nevertheless, at this stage, AM is not intended to replace conventional manufacturing technologies, but to work in tandem with them. AM is still in the development phase, and many areas still need to be explored. Products made with 3D printing need to be evaluated over the long term to assess their behavior in long-term use, from a mechanical properties point of view, but also for its environmental impact. For example, residues from conventional wood processing could be used as materials for 3D printing, adding value to low-grade residues that are now often used as fuel or even sent to landfills. Even used wood products at the end of their life could be used as raw materials for additive manufacturing technologies. To achieve better mechanical properties of 3D-printed parts, the use of wood fibers, nanocellulose and continuous fibers printing needs to be further explored. There are also several possibilities to increase use of wood and additive manufacturing in the construction industry. In this way, even greater use of wood and its positive impact on the environment could be achieved.

## Figures and Tables

**Table 1:** Most promising areas of 3D-printing technologies and applications of wood and wood components.

Application	3DP Technology	Matrix	Wood Content (%), Used Component	Reference
Use of wood as a filler	FDM	PLA	5	[6]
		PLA	12.5–25	[32]
		PLA	5–20	[15]
		PLA	10–50	[31]
		PLA	20	[98]
		TPU	20	[99]
		PLA/PHA	30	[33]
		ABS	19–39	[67]
	DDM	starch	66	[88]
	LDM	PVAc/UF	15–25	[51]
	LDM	gypsum, methyl cellulose and cement	67–85	[39]
	LDM	methylcellulose	84.5–89	[50]
3D-printing constructions	BAAM	PLA	20	[56]
	LOM	PVA on top		[48]
Use of recycled furniture in printing materials	FDM	PLA	10–40	[92]
Continuous fiber printing	FDM+continuous fiber	Flax fiber/PLA		[58] *
	FDM+continuous fiber	twisted yarns of natural jute fibers/PLA		[57] *
Mycelium composites	paste material extrusion FDM+paste material extrusion	Mycelium Wood particles		[71] [70] [69]
3D printing in furniture production	FDM	PLA		[62]
Use of wood components	FDM	PLA	1, nanocellulose	[34]
	Liquid ink printing/extrusion		2–3.3, nanocellulose	[100]
			5–11, xylan	[100]
	FDM	PLA	5–15, lignin	[37]
	FDM	PLA	20–30, lignin	[16]
		PLA	20, tannin	[101]
4D printing	FDM	PLA+PHA	15	[102]
	FDM	PLA	15, 25	[76]
	FDM	PLA	20, 40	[77]

* Not wood fibers, but fibers from lignocellulosic plants (jute, flax fibers).

## Data Availability

Not applicable.
